# Overlapping and differential roles of plasma membrane calcium ATPases in Arabidopsis growth and environmental responses

**DOI:** 10.1093/jxb/ery073

**Published:** 2018-03-01

**Authors:** Huiyun Yu, Jiapei Yan, Xiangge Du, Jian Hua

**Affiliations:** 1 Research Center of Organic Agriculture Technology, College of Plant Protection, China Agricultural University, Beijing, PR China; 2 School of Integrative Plant Science, Plant Biology Section, Cornell University, Ithaca, NY, USA

**Keywords:** ACA, Arabidopsis, calcium, calcium pump, growth, immunity, stomatal movement

## Abstract

Plant cells have multiple plasma membrane (PM)-localized calcium ATPases (ACAs) pumping calcium ions out of the cytosol. Although the involvement of some of these ACAs in plant growth and immunity has been reported, their individual and combined functions have not been fully examined. Here, we analysed the effects of single and combined mutations of four ACA genes, *ACA8*, *ACA10*, *ACA12*, and *ACA13*, in a number of processes. We found that these four genes had both overlapping and differential involvements in vegetative growth, inflorescence growth, seeds setting, disease resistance and stomatal movement. Disruption of any of these four genes reduces seed setting, indicating their contribution to the overall fitness of the plants. While ACA10 and ACA8 play major roles in vegetative growth and immunity, ACA13 and ACA12 are also involved in these processes especially when the function of ACA10 and/or ACA8 is compromised. The loss of ACA13 and ACA10 function in combination with a reduction in function of ACA8 leads to seedling death at bolting, revealing the essential role of their collective function in plant growth. Taken together, this study indicates a highly tuned calcium system involving these PM-localized calcium pumps in plant growth and environmental responses.

## Teaser

Four plasma membrane calcium ATPases in Arabidopsis, ACA8, ACA10, ACA12, and ACA13, have overlapping and differential roles in vegetative growth, reproductive development, stomatal movement control, and disease resistance.

## Introduction

Calcium (Ca^2+^) is an essential second messenger for cellular signal transduction and has a wide range of physiological roles in response to various environmental stimuli such as light, temperature, and pathogens (Sanders *et al.*, 2002; McAinsh and Pittman, 2009; Dodd *et al.*, 2010; Kudla *et al.*, 2010). It has been hypothesized that calcium carries information on the stimulus specifically through the amplitude, frequency, and duration of calcium spikes (Dodd *et al.*, 2010; Bonza and De Michelis, 2011). This Ca^2+^ signature is shaped by the combination of activities of membrane transport proteins in Ca^2+^ influx and Ca^2+^ efflux systems. Calcium influx is mediated by a number of ion channels such as Ca^2+^-permeable cyclic nucleotide-gated channels and voltage-gated channels (Kudla *et al.*, 2010). These channels reside at the plasma membrane (PM), endoplasmic reticulum (ER), vacuole, or mitochondria and are thought to collectively contribute to the dynamics of calcium signals (Geisler *et al.*, 2000*a*; Sze *et al.*, 2000; Boursiac and Harper, 2007). Ca^2+^ efflux requires cotransport systems and energy-dependent Ca^2+^ pumps such as the ER-type Ca^2+^-ATPases (ECAs) and the autoinhibited Ca^2+^-ATPases (ACAs) (Steinhorst and Kudla, 2013). Fourteen Ca^2+^ pumps have been found in Arabidopsis, ten of which are ACAs and four are ECAs. Based on the sequence similarity and intron positions of their genes, ACA proteins can be classified into four clusters that appear to be conserved in flowering plants (Baxter *et al.*, 2003; Boursiac and Harper, 2007; Bonza and De Michelis, 2011). Cluster 1 consists of ER-localized ACA1 and ACA2 as well as PM-localized ACA7 (Hong *et al.*, 1999; Dunkley *et al.*, 2006; Lucca and León, 2012). Cluster 2 consists of vacuole-localized ACA4 and ACA11 (Geisler *et al.*, 2000*b*; Boursiac *et al.*, 2010). Cluster 3 consists of PM-localized ACA12 and ACA13, both of which are encoded by intron-less genes (Iwano *et al.*, 2014; Limonta *et al.*, 2014). Cluster 4 consists of PM-localized ACA8, ACA9, and ACA10, which are characterized by a slightly larger molecular mass mainly due to a longer N-terminal domain (Bonza *et al.*, 2000; Schiøtt *et al.*, 2004; George *et al.*, 2008).

Ca^2+^ pumps are considered to be crucial in regulating the shape of the calcium transient, after cytosolic Ca^2+^ concentration is elevated by stimuli, during the recovery of basal cytosolic level (Dodd *et al.*, 2010; Kudla *et al.*, 2010; Steinhorst and Kudla, 2013; Costa *et al.*, 2017). A number of plant ACA genes have been characterized as Ca^2+^ pumps by their expressions in the yeast mutant strain K616, which lacks endogenous Ca^2+^-ATPases (Curran *et al.*, 2000; Bonza *et al.*, 2004; Baekgaard *et al.*, 2006). The N-terminus of several ACAs contains an auto-inhibitory domain that inhibits the activity of the ATPase domain to keep the pump activity low (Giacometti *et al.*, 2011; Costa *et al.*, 2017). This auto-inhibitory domain overlaps with the calmodulin (CaM)-binding motifs, and calmodulin binding is thought to release auto-inhibition and therefore activate calcium ATPase (Tidow *et al.*, 2012; Costa *et al.*, 2017). When the auto-inhibitory domain is deleted, ACA10 becames ‘deregulated’ and complements the calcium pump deficiency of K616 while the full-length ACA10 does not (Schiøtt and Palmgren, 2005). Similar pump activities have been found in yeasts for the deregulated (N-terminus deleted) versions of ACA8, ACA9, and ACA13 (Bonza *et al.*, 2004; Schiøtt and Palmgren, 2005; Iwano *et al.*, 2014). However, ACA12 appears to be a deregulated pump (Limonta *et al.*, 2014); unlike other ACAs, it is not stimulated by CaM although it could bind to CaM, and a full-length ACA12 rescues the defect of K616. This is likely due to the lack of two amino acidic residues that are conserved in other subgroups of ACAs (Limonta *et al.*, 2014).

ACA proteins localized on the PM are implicated in growth and development regulation in plants. Several PM-localized ACAs, ACA7, ACA9, and ACA13, have been shown to be important for pollen development and/or pollen function. The *ACA9* gene is expressed specifically in pollen, and its loss-of-function (LOF) mutant displays reduced growth of pollen tubes and a high frequency of aborted fertilization (Schiøtt *et al.*, 2004). The LOF mutant of *ACA7* has dead pollen grains in mature flowers (Lucca and León, 2012). The ACA13 protein localizes to the PM and vesicles and accumulates at the pollen tube penetration site after pollination; its LOF mutant has a pollination defect (Iwano *et al.*, 2014). These results indicate a critical role of Ca^2+^ efflux at the PM in reproductive growth. ACA10 and ACA8 are implicated in promoting growth of rosettes and inflorescences (Bonza *et al.*, 2000; George *et al.*, 2008; Yang *et al.*, 2017). The *ACA10* LOF mutant in the No-0 background has a compact inflorescence stem (George *et al.*, 2008) and the *aca10 aca8* double mutant has much reduced growth of the rosette and inflorescence (Yang *et al.*, 2017).

PM-localized ACAs are also involved in plant stress response regulation. ACA8 was reported to be associated with FLS2, a receptor for the pathogen pattern flg22 (dit Frey *et al.*, 2012). The *aca10* LOF mutant in No-0 has higher resistance to the bacterial pathogen *Pseudomonas syringae* pv. *tomato* (*Pst*) DC3000, and so does the *aca8 aca10* double mutant in Col-0 (Yang *et al.*, 2017). Defense genes are up-regulated in these mutants even under non-infection conditions, and reducing the defense responses alleviates the growth defect in these mutants (Yang *et al.*, 2017). Interestingly, ACAs on the vacuole are also implicated in plant immunity (Boursiac *et al.*, 2010). The double LOF mutant of vacuole-localized ACA4 and ACA11 displays a high frequency of hypersensitive response-like lesions associated with up-regulation of the salicylic acid pathway and enhanced disease resistance (Geisler *et al.*, 2000*b*; Boursiac *et al.*, 2010). Stomatal movement in response to pathogen has also been shown to be modulated by PM-localized ACAs. The *aca8 aca10* double mutant does not close its stomata in response to bacterial pathogen as the wild type does (Yang *et al.*, 2017). In Arabidopsis leaves and roots, ACA8 is also involved in the response to wounding-related signals (Costa *et al.*, 2017).

The role of ACAs in growth and immunity is likely due to their impact on the calcium signal or calcium level in cellular compartments. ACA8 and ACA10 are implicated in calcium signature generation. External calcium application induces cytosolic calcium oscillations in the wild type guard cells, and the loss of either ACA8 or ACA10 function abolishes the oscillations in the cytosol (Yang *et al.*, 2017). In addition, the *aca8 aca10* double mutant inoculated with the bacterial elicitor flagellin exhibits a lower cytosolic Ca^2+^ transient increase than the wild type (dit Frey *et al.*, 2012).

Despite of these studies, we still do not fully understand the roles of PM-localized ACAs as a whole or individually even in the model plant Arabidopsis. These proteins might differ in their biochemical activities and regulation. ACA8, ACA10, and ACA13 are thought to have the auto-inhibitory domain while ACA12 likely does not (Iwano *et al.*, 2014; Limonta *et al.*, 2014). Potential genetic redundancy, full or partial, might mask the role of ACAs expressed in the same cell and the same compartment in single mutant studies. In addition, loss of one member’s function might be compensated by up-regulation of another member with similar expression pattern and subcellular localization. ACA12 and ACA13 were both found to have a higher expression in the *aca8 aca10* mutant than in the wild type under flagellin treatment (dit Frey *et al.*, 2012). Here we investigated the role of four PM-localized ACAs, namely ACA12 and ACA13 along with ACA8 and ACA10, in a number of processes. Analysis of mutant combinations of the four *ACA* genes reveals their overlapping and differential involvement in development and defense responses. These PM-localized calcium pumps may contribute to a finely tuned calcium signalling system in plant growth and immunity.

## Materials and methods

### Plants and growth conditions

Mutants of *ACA8* (GK-688H09), *ACA10* (GK-044H01), *ACA12* (SALK_098383), and *ACA13* (SAIL_878_B06) were as previously described (dit Frey *et al.*, 2012; Iwano *et al.*, 2014; Limonta *et al.*, 2014). For generating mutant combinations, *aca12* was crossed with *aca13*; *aca12* and *aca13* were crossed with *aca8 aca10*. Mutants *aca12 aca13*, *aca8 aca12*, *aca8 aca13*, *aca10 aca12*, *aca10 aca13*, *aca8 aca10 aca12*, *aca8 aca10 aca13/+*, and *aca10 aca13 aca8/+* were isolated in the F2 populations by PCR (all primers used are summarized in Supplementary Table S1 at *JXB* online).

The Arabidopsis plants were grown in soil with light intensity at 100 μmol m^−2^ s^−1^ and relative humidity at 50–70%. Constant light conditions were used for growth phenotype analysis and gene expression analysis unless specified otherwise. Plants were grown under 12 h light/12 h dark for pathogen resistance assay and stomatal assay unless specified otherwise.

Hydroponic culture was performed as previously described with slight modification (Boursiac *et al.*, 2010). Arabidopsis seeds were sown on plates on half-strength Murashige and Skoog medium complemented by 1% Suc and 7 g l^−1^ agar. After 2 d in the dark at 4°C, seeds were germinated under 24 h light at 22°C. Five-day-old seedlings were transferred onto a floating foam support in 300 ml boxes covered by foil and filled with a standard hydroponic solution of 1.25 mM KNO_3_, 0.75 mM MgSO_4_, 1.5 mM Ca(NO_3_)_2_, 0.5 mM KH_2_PO_4_, 50 mM FeEDTA, 50 mM H_3_BO_3_, 12 mM MnSO_4_, 0.7 mM CuSO_4_, 1 mM ZnSO_4_, 0.24 mM MoO_4_Na_2_, and 100 mM Na_2_SiO_3_. Hydroponic solutions were replaced every week. For suppression conditions, the standard hydroponic solution above was supplemented with 15 mM NH_4_NO_3_ to a final concentration of NO_3_^−^ at 19.25 mM.

### Quantitative real-time quantitative PCR and gene expression

Total RNA was extracted from soil-grown 15-day-old plants with TRIzol reagent (Invitrogen) according to the manufacturer’s protocol. The cDNAs were synthesized from total RNA using AffinityScript QPCR cDNA Synthesis Kit (Agilent Technologies). Real-time quantitative PCR was performed with the Bio-Rad PCR System using iQSYBR GREEN SuperMix (Bio-Rad). Quantitative real time PCR (qRT-PCR) was performed using primers PR1-QRT-F and PR1-QRT-R to test the expression of *PR1*, and primers ACT2 F and ACT2 R to amply *ACTIN2*. All primers are listed in Supplementary Table S1. The relative expression level was calculated by the ${\text{2}}_{}^{-\Delta \Delta C_{\text{T}}}$ method (Livak and Schmittgen, 2001) with three biological replicates. For each biological replicate, there were three technical replicates. *ACTIN2* was used as a reference gene for qRT-PCR.

### Pathogen resistance assay

The bacteria strain *Pst* DC3000 was grown for 2 d on King’s B medium and resuspended at 5 × 10^6^ colony forming units ml^−1^ (OD_600_=0.05) in a solution of 10 mM MgCl_2_ and 0.02% (v/v) Silwet L-77. Two-week-old seedlings grown at 22°C or 12-day-old seedlings grown at 28°C were dipping-inoculated with bacterial solution and kept covered for 1 h. The amount of bacteria in the plants was analysed at 1 h and 3 d after dipping (0 and 3 d post-innoculation, respectively). The aerial parts of three inoculated seedlings were pooled as one sample, and three samples were collected for each genotype and time point. Seedlings were ground in 1 ml of 10 mM MgCl_2_, and serial dilutions of the ground tissue were used to determine the number of colony forming units (log_10_) per mg of fresh leaf tissues. For spray-inoculation, the resuspended bacteria were sprayed on plants until all leaves were wet.

### Measurement of leaf physiological parameters

Rosettes from 35-day-old plants were weighed as biomass. Rosettes were also photographed from the top, and the diameter of the rosette was defined as the diameter of the smallest circle that covers the whole rosette using ImageJ software. Thirty plants were measured for each genotype/condition.

### Stomatal closure assay

Stomatal closure assays were performed as previously described (Zeng *et al.*, 2010; Gou *et al.*, 2015) with slight modifications. Plants were grown under a 12/12 h photoperiod at 22°C for ABA- or Ca^2+^-induced stomatal closure assay. Leaves were collected at 4 weeks after germination and placed in MES buffer (10 mM MES–Tris, pH 6.15, 50 mM KCl) or MES buffer with 20 μM abscisic acid (ABA) or MES buffer with 100 μM CaCl_2_ in closed Petri dishes for 1.5 h. Epidermises were peeled and imaged with a Lecia ICC50HD microscope. At least 30 stomatal apertures were measured for each sample from about five expanded leaves using ImageJ software. Each experiment was repeated at least three times.

### Statistical analysis

Rosette morphology (weight and diameter), number of seeds in siliques, pathogen resistance assay and stomatal assay data were subjected to a one-way analysis of variance (ANOVA) followed by Duncan’s new multiple range test to assess differences between different genotypes. *P* was calculated at a 5% significance level to allow easy comparison of differences.

## Results

### Expression patterns of *ACA8*, *ACA10*, *ACA12*, and *ACA13*

**Fig. 1.**
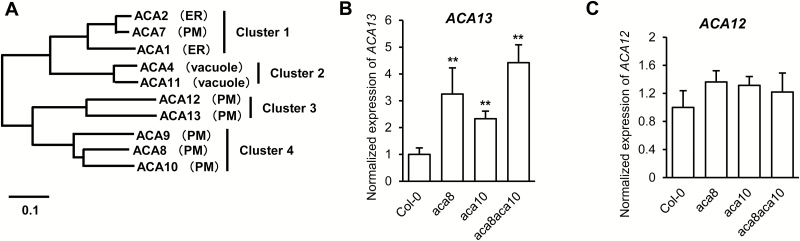
Expression patterns of *ACA12* and *ACA13*. (A) Phylogenetic tree of ACAs modified from Iwano *et al.* (2014). ER, endoplasmic reticulum; PM, plasma membrane. (B, C) Expression of *ACA13* (B) and *ACA12* (C) analysed by qRT-PCR in leaves of wild-type Col-0, *aca8*, *aca10*, and *aca8 aca10* at 2 weeks old grown at 22°C with 12 h light. Data are presented as means ±SD for three independent biological replicates. **Significant differences between Col-0, *aca8*, *aca10* and *aca8 aca10* at *P*<0.01 based on one-way ANOVA followed by Duncan’s new multiple range test.

ACA12 and ACA13 are the only two members in cluster 3, which is most closely related with cluster 4 that consists of PM-localized ACA8, ACA9, and ACA10 (George *et al.*, 2008; Iwano *et al.*, 2014) (Fig. 1A). We examined the tissue expression pattern of these PM ACAs through the public RNA-seq and microarray data (Winter *et al.*, 2007). *ACA10* and *ACA8* have a higher expression than *ACA12* and *ACA13* in most tissues and developmental stages, including leaves, roots, young flowers (stages 9–11), and seeds (see Supplementary Table S2A). However, in the stamen at flower stage 15, *ACA13* has a much higher expression than the other three *ACAs* (Supplementary Table S2A). In mature pollens, *ACA8* has a slightly higher expression than the other three *ACA*s (Supplementary Table S2A). In guard cells, *ACA12* has the highest expression and *ACA8* has the lowest expression (Supplementary Table S2A). These genes also showed differential expression in response to biotic and abiotic stresses (Supplementary Table S2B, C). *ACA10* was induced to a greater extent than *ACA8* in response to *Botrytis cinerea*, *Pseudomonas syringae*, *Phytophthora infestans*, *Erysiphe orontii*, and pathogen pattern flg22 (Supplementary Table S2B). *ACA12* was also highly induced by these pathogens or pathogen signals except for *Erysiphe orontii,* with the highest fold induction among the four genes, while no induction was seen for *ACA13* except in response; to *Pseudomonas syringae* (Supplementary Table S2B). The expression of these four genes did not seem to be responsive to many abiotic stresses, except that *ACA8* was induced by cold, *ACA10* and *ACA12* were induced by UV-B, and *ACA13* was induced by osmotic stress (Supplementary Table S2C). Therefore, the tissue specificity pattern and environmental response of their RNA expressions are different among the four *ACAs*.

*ACA12* and *ACA13* were postulated to compensate for the loss of *ACA8* and *ACA10* as their expressions after flg22 treatment were higher in the *aca8 aca10* double mutant than the wild type (dit Frey *et al.*, 2012). To examine this further, we analysed the expression of *ACA12* and *ACA13* in the single and double mutants of *aca8* and *aca10* under normal growth conditions without the treatment of the pathogen signal flg22. qRT-PCR revealed that *ACA13* was up-regulated in leaves of the single *aca8* and *aca10* mutants compared with the wild type by ~2.2- and ~1.4-fold, respectively (Fig. 1B). The transcript abundance of *ACA13* in leaves was increased further in the *aca8 aca10* double mutant by ~3.0-fold in comparison to the wild type (Fig. 1B). The expression of *ACA12* was slightly increased in the single and double *aca8* and *aca10* mutants by ~0.5-fold compared with the wild type (Fig. 1C), but this increase was not significant statisticallly. This expression pattern suggests that *ACA13* and perhaps *ACA12* might play compensatory roles for *ACA8* and *ACA10* even under non-pathogen-invasion conditions.

### The role of *ACA*s in vegetative growth

To identify the biological roles of *ACA12* and *ACA13*, we analysed mutants of *ACA12* (SALK_098383) and *ACA13* (SAIL_878_B06) from the T-DNA insertion line collections (Alonso *et al.*, 2003; Iwano *et al.*, 2014). These mutants are LOF because T-DNAs were inserted in the only exon and RT-PCR study revealed no expression of *ACA12* and *ACA13* genes in their respective mutants (Iwano *et al.*, 2014).

**Fig. 2.**
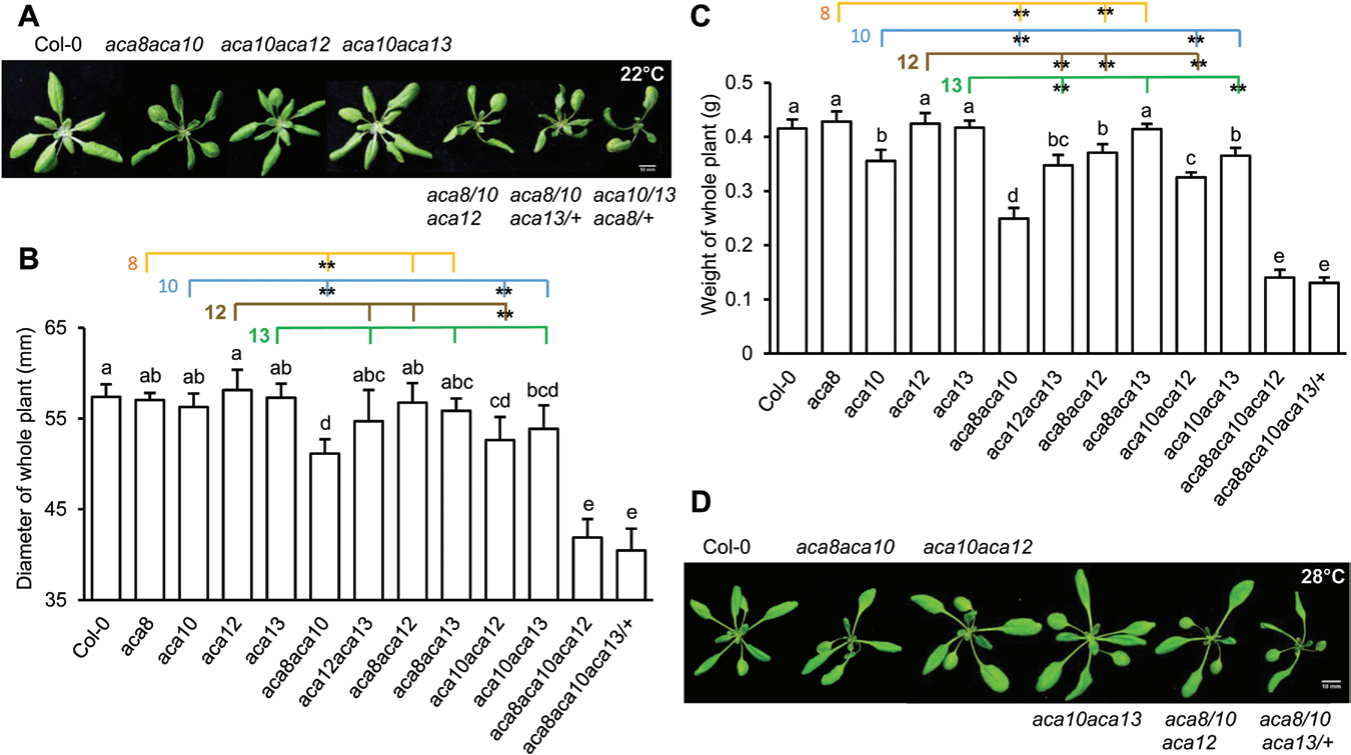
Growth phenotypes of the *aca* mutants in the vegetative stage. Shown are growth phenotypes under constant light at 22°C (A, B, C) and 28°C (D). (A) Rosette leaves of 18-day-old plant after germination at 22°C. (B, C) Quantification by diameter (mm) (B) and weight (C) of rosettes at 21 d after germination at 22°C. Data shown are means ±SD (*n*=24). Each colored line links a single *aca* mutant with double mutants containing that single mutation. Letters indicate statistically significant differences for different genotypes determined by one-way ANOVA (*P*<0.05) followed by Duncan’s new multiple range test. **Significant difference (*P*<0.01) between a double mutant and that single mutant, calculated from one-way ANOVA followed by Duncan’s new multiple range test . (D) Rosette leaves of 15-day seedlings at 28°C.

To test the potential genetic redundancy among the four *ACA* genes, we generated all six double mutants among *aca8*, *aca10*, *aca12*, and *aca13*, namely *aca8 aca10*, *aca12 aca13*, *aca8 aca12*, *aca8 aca13*, *aca10 aca12*, and *aca10 aca13*. At the vegetative stage, none of the single mutants grew drastically differently from the wild type at 22 and 28°C (Fig. 2A, D; Supplementary Fig. S1A, B). Subtle differences were observed when the mutants were quantified by their size and weight at the 3-week-old stage grown under 22°C (Fig. 2B, C). For diameter of rosettes, none of the single mutants showed a significant difference compared with the wild type (Fig. 2B). For weight of whole plant, the *aca10* mutant was lighter than the wild type at the vegetative stage (Fig. 2C). Therefore, the loss of *ACA8*, *ACA12*, or *ACA13* alone did not cause obvious growth defects, suggesting a minor role or overlapping role of these two genes with others in the development of rosettes (see Supplementary Fig. S2A).

We then analysed the growth phenotypes of all these six double mutant combinations. The *aca8 aca10* double mutant showed the largest visible growth defects compared with the wild type among the six double mutants. Young leaves of *aca8 aca10* were more compact and the mature leaves were narrower with leaf blades twisted downward compared with the wild type at 22°C (Fig. 2A; Supplementary Fig. S1A). When quantified by the diameter of the rosette size at vegetative stage, *aca10 aca12* and *aca8 aca10* were significantly smaller than their respective single mutants (Fig. 2B). Therefore, double mutants containing *aca10* exhibited the strongest growth defect, indicating a major role of *ACA10* in rosette growth. When biomass was measured, four out of the six double mutants, *aca8 aca10*, *aca12 aca13*, *aca8 aca12*, and *aca10 aca12*, were all lighter than their respective single mutants (Fig. 2B, C). Only the double mutant *aca8 aca13* did not exhibit significant differences from the wild type or single mutants (Fig. 2C). When plants grew at 28°C, all growth defects in the double mutants were abolished, except for *aca8 aca10*, which still showed a more compact phenotype at seedling stage (Fig. 2D; Supplementary Fig. S1B). Comparison of strength of defects in the double mutants indicates that *ACA10* has a major role in vegetative growth followed by *ACA8*, and then *ACA12* and *ACA13* (see Supplementary Fig. S2A, B).

Because the *aca8 aca10* double mutant exhibited the most severe defect among double mutant combinations, we examined the role of *ACA12* and *ACA13* in the *aca8 aca10* double mutant background. By crossing *aca12* and *aca13* single mutants with the *aca8 aca10* double mutant, we obtained the triple mutant *aca8 aca10 aca12* but not *aca8 aca10 aca13* due to seed abortion (see below). All the *aca8 aca10 aca12*, *aca8 aca10 aca13/+*, and *aca8/+ aca10 aca13* triple mutants showed more severe growth defects than the *aca8 aca10* double mutant at 22°C (Fig. 2A; Supplementary Fig. S1A). They had narrower leaves, much smaller rosette size, and less biomass (Fig. 2A–C). The *aca8 aca10 aca12* and *aca8 aca10 aca13/+* mutants exhibited a less severe growth defect at 28°C than at 22°C, and they had a similar morphology to the *aca8 aca10* double mutant at 28°C (Fig. 2A, D; Supplementary Fig. S1). Therefore, the function of *ACA12* and *ACA13* was revealed in the *aca8 aca10* double mutant background.

### The role of *ACA*s in inflorescence growth

**Fig. 3.**
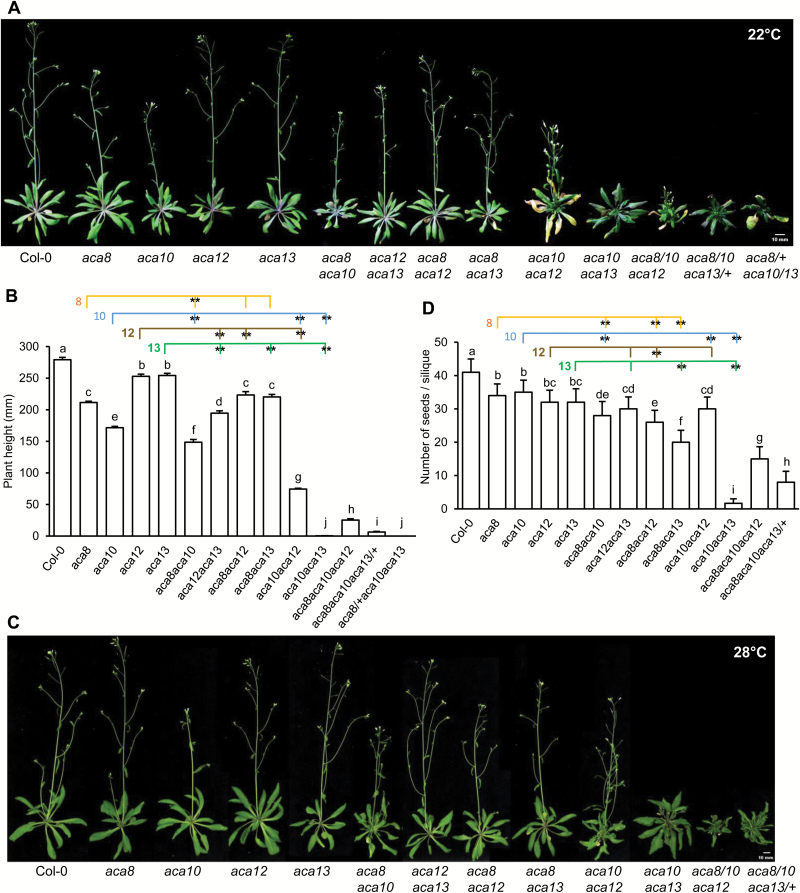
Growth phenotypes of the *aca* mutants at the flowering stage. Shown are growth phenotypes under constant light at 22°C (A, B, D) and 28°C (C). (A) Inflorescence and rosette phenotypes of the wild type Col-0 and mutant plants at 30 d after germination at 22°C. (B) Inflorescent height at 30 d after germination. Data shown are means ±SD (*n*=6). Each colored line links a single *aca* mutant with double mutants containing that single mutation. Letters indicate statistically significant differences for different genotypes determined by one-way ANOVA (*P*<0.05) followed by Duncan’s new multiple range test. **Significant difference (*P*<0.01) between a double mutant and that single mutant, calculated from one-way ANOVA followed by Duncan’s new multiple range test. (C) Inflorescence and rosette phenotypes of the wild type Col-0 and mutant plants at 30 d after germination at 28°C. (D) Number of seeds per silique after pollination. Data shown are means ±SD (*n*=25). All annotations are the same as in (B).

After bolting, these mutants exhibited growth defects of a similar extent to those of the early seedling stages. At 22°C, the *aca10* mutant had the shortest inflorescence among the four single mutants and more axillary stems than the wild type, while single mutants of *ACA8*, *ACA12*, and *ACA13* also exhibited obvious difference compared with the wild type (Fig. 3A, B). Overlapping functions were found for *ACA10* with *ACA8*, *ACA12*, and *ACA13* in inflorescence development as in vegetative rosette development (see Supplementary Fig. S2C). Each of the *aca8* and *aca12* single mutations enhanced the inflorescence growth defect of *aca10*, and the *aca8 aca10* and *aca12 aca10* double mutants had shorter inflorescence stems than *aca10* (Fig. 3A, B). The *aca13* mutation had the largest effect when combined with *aca10*, and the *aca10 aca13* mutant had the most reduced inflorescence stem among all double mutants with *aca10* (Fig. 3A, B), indicating the dominant role of *ACA10* in inflorescence growth. All the double mutants had reduced inflorescence height compared with the wild type (Fig. 3A, B). The severity of the double mutants indicates that the major role of *ACA10* is followed by that of *ACA13*, *ACA8*, and lastly *ACA12* in this process (Supplementary Fig. S2C). The overlapping function was also evident in the triple mutants. The *aca8 aca10 aca13/+* triple mutant showed a more severe phenotype than *aca8 aca10*, and the *aca8/+ aca10 aca13* triple mutant never bolted and died at the wild type bolting stage (Fig. 3A, B).

The *aca10* single mutant also showed reduced inflorescence height compared with the wild type when grown at 28°C, and a more severe defect was observed in the double mutants *aca8 aca10*, *aca10 aca12* and *aca10 aca13* (Fig. 3C). Especially, *aca10 aca13* showed inflorescence internodes much shorter than those of the *aca10* single mutant at 28°C, similar to that at 22°C (Fig. 3A, C). In addition, the *aca8 aca12* and *aca8 aca13* double mutants had a short inflorescence, while the *aca8 aca10 aca12* and *aca8 aca10 aca13/+* triple mutants had almost no noticeable inflorescence elongation (Fig. 3C). Together, these data indicate that *ACA10* plays an important role in inflorescence development (see Supplementary Fig. S2C), and the function of *ACA12* and *ACA13* can be revealed in the *aca8 aca10* double mutant background.

### The role of *ACA*s in seed setting

Because *ACA13* has a critical function for successful pollination (Iwano *et al.*, 2014), we examined the roles of these four ACAs in reproductive growth by examining the number of seeds per silique of *aca* mutant combinations. For all single mutants, the number of seeds per silique was slightly but significantly less than that in the wild type (Fig. 3D), indicating that all four ACAs could impact seed setting. Among the double mutants, *aca10 aca13* exhibited the most severe reduction of seed number, and had almost no seeds set in its siliques (Fig. 3D). This suggests that *ACA13* and *ACA10* play major roles in seed setting. *ACA13* appears to play a larger role than *ACA10*, as the double mutant *aca8 aca13* had less seed setting than *aca8 aca10* (Fig. 3D). *ACA12* had a minor role comapred with *ACA8* as the *aca8* double mutants had less seed setting than the respective *aca12* doubles with *aca13* or *aca10* (Fig. 3D). These results indicate that all four ACAs have functions in reproduction. *ACA13* has a major role and *ACA10* and *ACA8* have overlapping functions with *ACA13* (see Supplementary Fig. S2D). Interestingly, the *aca8 aca10 aca13/+* triple mutant had less seed production than *aca8 aca10* (Fig. 3D), suggesting that seed setting is also very sensitive to the level of ACA activity.

### The role of *ACA*s in disease resistance

**Fig. 4.**
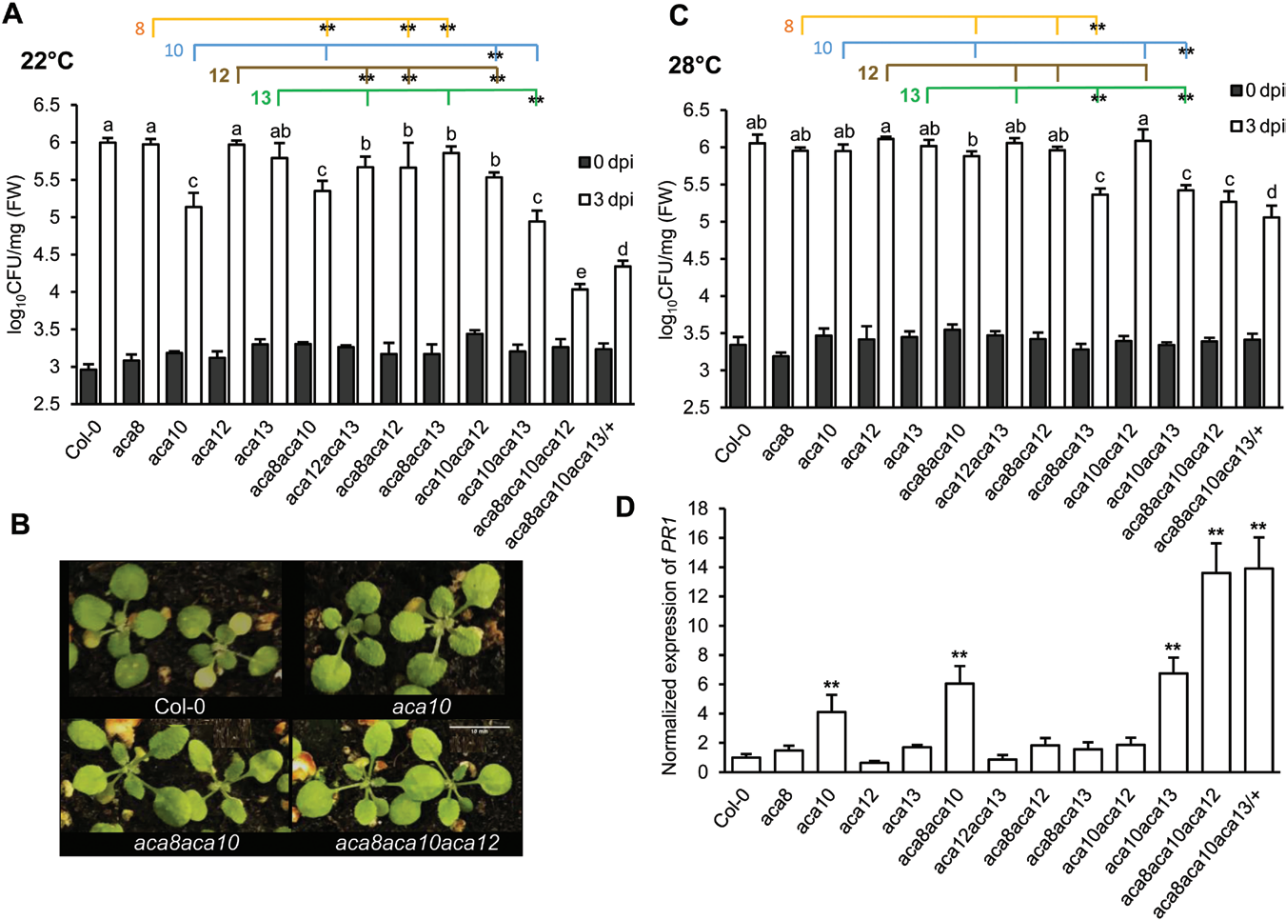
Plant immunity of the *aca* mutants. (A) Growth of *Pst* DC3000 in the wild type Col-0 and mutant plants at 0 and 3 d post-inoculation (dpi) at 22°C. Values represent means ±SD for three independent experiments (*n*=3). Letters indicate statistically significant differences for different genotypes determined by one-way ANOVA (*P*<0.05) followed by Duncan’s new multiple range test. FW, fresh weight. Each colored line links a single *aca* mutant with double mutants containing that single mutation. **Significant difference (*P*<0.01) between a double mutant and that single mutant, calculated from one-way ANOVA followed by Duncan’s new multiple range test. (B) Disease symptoms in the wild type and *aca* mutant plants at 22°C after spray inoculation with *Pst* DC3000. (C) Growth of *Pst* DC3000 in the wild type Col-0 and mutant plants at 0 and 3 d post-inoculation at 28°C. All annotations are the same as in (A). (D) qRT-PCR analysis of *PR1* expression in the wild type Col-0 and mutant plants. Data are means ±SD for three independent replicates. Shown are data from one representative experiment. **Significant differences (*P*<0.01) between Col-0 and *aca* mutants based on Student’s *t*-test.

To examine the possible roles of *ACA12* and *ACA13* in plant immunity, we analysed pathogen growth in the *aca* mutant plants. Inoculated by the dipping method, the *aca10* mutant, but not other single mutants, supported significantly less growth of the virulent pathogen *Pseudomonas syringe* pv *tomato* (*Pst*) DC3000 compared with the wild type at 22°C (Fig. 4A), which is consistent with the previous report (Yang *et al.*, 2017). We further analysed resistance of double mutants to the virulent pathogen *Pst* DC3000. Double mutants combined with *aca10*, including *aca8 aca10* and *aca10 aca13*, exhibited an enhanced resistance similarly to the *aca10* single mutants, and *aca10aca12* had a slightly reduced resistance than the *aca10* mutant (Fig. 4A). None of the other double mutants without *aca10* had enhanced resistance compared with their respective single mutants, indicating a major role of *ACA10* in plant immunity (Fig. 4A; Supplementary Fig. S2E). The role of *ACA12* and *ACA13* in immunity was revealed in the absence of both *ACA10* and *ACA8* as the *aca8 aca10 aca12* and *aca8 aca10 aca13/+* triple mutants showed even higher resistance to *Pst* DC3000 than the *aca10* or *aca8 aca10* mutants (Fig. 4A). This observation was corroborated by resistance assay using a different method, spray inoculation. After being sprayed with *Pst* DC3000, the wild type displayed yellow diseased areas on most of the true leaves besides yellowing of the cotyledons (Fig. 4B). In contrast, *aca10* and *aca8 aca10* displayed less yellowing in true leaves and *aca8 aca10 aca12* had no yellowing in true leaves (Fig. 4B). Therefore, *ACA12* and *ACA13* could have overlapping functions with *ACA8* in the absence of *ACA10* in resistance against *Pst* DC3000 (see Supplementary Fig. S2E).

We subsequently analysed the pathogen growth at 28°C because disease resistance is often suppressed by a high growth temperature (Hua, 2013). Indeed, the increased resistance exhibited at 22°C was not observed for *aca10* or its double mutants at 28°C, except for *aca10 aca13* (Fig. 4C). Interestingly, the *aca8 aca13* double mutant exhibited an enhanced resistance at 28°C compared with the wild type or the single mutants while it only exhibited a slightly enhanced resistance at 22°C compared with the single mutants (Fig. 4A, C). This suggests that *ACA13* may have overlapping functions with *ACA10* and *ACA8* in repressing defense even at 28°C (see Supplementary Fig. S2F). This notion is supported by the enhanced resistance exhibited in the *aca8 aca10 aca13/+* triple mutants compared with the *aca8 aca10* double mutant (Fig. 4C).

We further analyzed the expression of a defense response gene, *PR1*, in the wild type and all mutant plants by qRT-PCR. Among the single mutants, *PR1* was up-regulated only in the *aca10* mutant that exhibited enhanced resistance (Fig. 4D). *PR1* was also up-regulated in the *aca8 aca10* and *aca10aca13* double mutants, but not other double mutants (Fig. 4D). The highest *PR1* expression was observed in *aca8 aca10 aca12* and *aca8 aca10 aca13/+* triple mutant plants (Fig. 4D). These results revealed a correlation of *PR1* expression level in the absence of pathogen with the resistance level to the pathogen, indicating a constitutive defense response in some *aca* mutants that likely leads to the enhanced disease resistance.

### The role of *ACA*s in stomatal movement

**Fig. 5.**
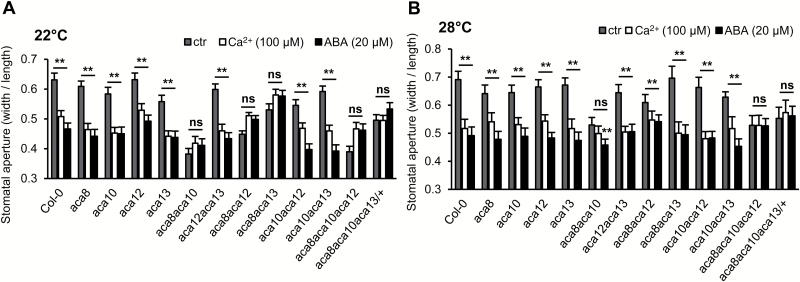
Stomatal closure responses in the *aca* mutants. (A, B) Stomatal apertures after ABA- and treatment at 22°C (A) and 28°C (B). Epidermal peels of the wild type Col-0 and mutant plants were pre-treated with opening buffer and stomatal apertures were measured before and after treatment with ABA and Ca^2+^. Each data point represents the mean ±SD (*n*=30). The experiments were repeated three times. Shown are data from one representative experiment. Each colored line links a single *aca* mutant with double mutants containing that single mutation. **Significant difference (*P*<0.01) between a double mutant and that single mutant, calculated from one-way ANOVA followed by Duncan’s new multiple range test.

A previous study indicates that *ACA8* and *ACA10* are positive regulators of stomatal closure in response to Ca^2+^ and a coronatine-deficient (COR^−^) *Pst* DC3000 strain (Yang *et al.*, 2017). We determined whether *ACA12* or *ACA13* is also involved in stomatal control. Incubation with 20 μM ABA or 100 μM Ca^2+^ induced stomatal closure in the wild type at 22°C (Fig. 5A), which was consistent with previous reports (Zeng *et al.*, 2010; Zou *et al.*, 2010). All single mutants were sensitive to ABA or Ca^2+^ in their stomatal closure response, similarly to the wild type (Fig. 5A). In contrast, ABA- and Ca^2+^-induced stomatal closure at 22°C was impaired in all *aca8* double mutants, including *aca8 aca10*, *aca8 aca12*, and *aca8 aca13* (Fig. 5A). These results suggest that *ACA8* has a major role in stomatal response and has an overlapping function with *ACA10*, *ACA12*, and *ACA13* (see Supplementary Fig. S2G). Not surprisingly, the *aca8 aca10 aca12* and *aca8 aca10 aca13/+* triple mutants also showed insensitivity to ABA and Ca^2+^ in the stomatal closure response (Fig. 5A). It was also noted that all mutants that were insensitive to ABA or Ca^2+^ in stomatal closure had smaller stomatal aperture compared with the wild type after being incubated with the opening buffer (Fig. 5A).

When plants were grown at 28°C, the stomatal closure in response to ABA or Ca^2+^ was significantly disrupted only in *aca8 aca10 aca12* and *aca8 aca10 aca13/+* triple mutants, much fewer than at 22°C (Fig. 5B). The *aca8 aca10* double mutant showed significant insensitivity to Ca^2+^ but not ABA (Fig. 5B). These three mutants, *aca8 aca10*, *aca8 aca10 aca12*, and *aca8 aca10 aca13/+*, exhibited smaller aperture after the opening buffer treatment at 28°C, similarly to 22°C (Fig. 5). Therefore, ABA or calcium insensitivity in the stomatal closure response at 22°C can be rescued by a temperature of 28°C in some mutants but not the more severe mutants such as *aca8 aca10 aca12* and *aca8 aca10 aca13/+* (see Supplementary Fig. S2H).

### Effect of anion supplements on the phenotypes of the *aca8 aca10* mutant

**Fig. 6.**
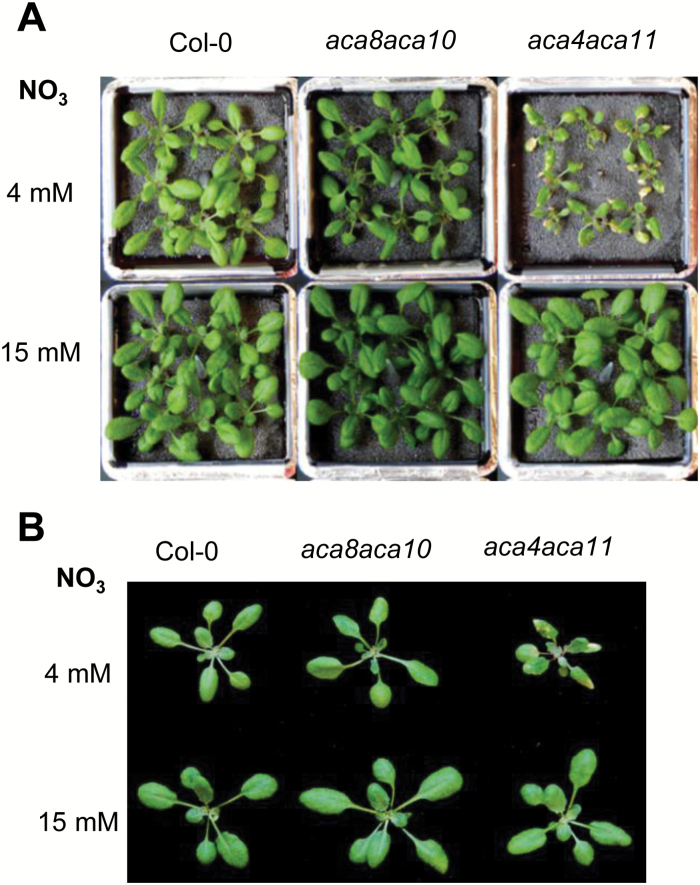
Effects of nutritional supplements on growth of *aca8 aca10* and *aca4 aca11*. (A, B) Fifteen-day-old seedlings grown in a hydroponic solution without or with supplemental 15 mM NH_4_NO_3_ after transfer from plate at 5 d of age; 4 mM NH_4_NO_3_: standard solution; 15 mM NH_4_NO_3_: standard solution supplemented with 15 mM NH_4_NO_3_.

In addition to temperature that was shown to modulate the *aca* mutant phenotype, an anion supplement was shown to suppress hypersensitive response-like necrotic lesions exhibited by the double mutant of vacuole ACAs *aca4 aca11* (Boursiac *et al.*, 2010). We therefore tested whether the mutant phenotypes of PM-localized ACAs might be suppressed by a similar anion addition. We used a standard hydroponic solution with NH_4_NO_3_ at 4 mM or supplemented to 15 mM NH_4_NO_3_ to grow the *aca8 aca10* double mutant. As reported previously (Boursiac *et al.*, 2010), the *aca4 aca11* mutant exhibited lesions in standard solution but not with 15 mM NH_4_NO_3_ (Fig. 6). Interestingly, the narrow leaf phenotype was suppressed in *aca8 aca10* double mutants by the addition of NH_4_NO_3_ (Fig. 6). In addition, the rosette size of *aca8 aca10* and *aca4 aca11* was comparable to the wild type when grown with the supplemental NH_4_NO_3_ while they were much smaller in the standard solution.

**Fig. 7.**
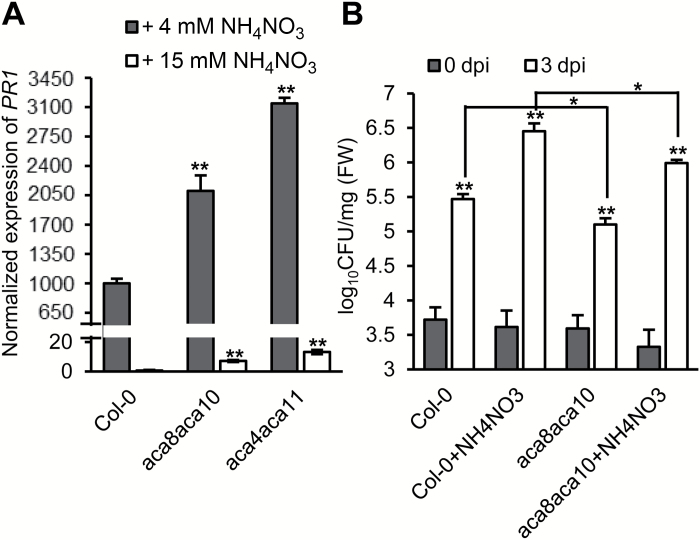
Effects of nutritional supplements on the immunity of Col-0, *aca8 aca10*, and *aca4 aca11*. (A) qRT-PCR analysis of *PR1* expression in Col-0, *aca8 aca10*, and *aca4 aca11* grown in two types of hydroponic solutions (4 mM NH_4_NO_3_: standard solution; 15 mM NH_4_NO_3_: standard solution supplemented with 15 mM NH_4_NO_3_). (B) Growth of *Pst* DC3000 in Col-0 and *aca8 aca10* at 0 and 3 dpi (+NH_4_NO_3_: standard solution supplemented with 15 mM NH_4_NO_3_). Asterisks indicate significant growth difference between two solutions or different genotypes (*P<0.05; **P<0.01), calculated from one-way ANOVA followed by Duncan’s new multiple range test. Data are means ±SD for three independent replicates. Shown are data from one representative experiment of two biologically independent experiments.

We tested whether the anion supplement could reduce defense response defects in the *aca8 aca10* mutant as observed in *aca4 aca11*. The *aca8 aca10* mutant exhibited a higher *PR1* expression, although not as high as *aca4 aca11*, when compared with the wild type (Fig. 7A). The NH_4_NO_3_ supplement significantly reduced the *PR1* transcript level in all plant lines including the wild type, and both *aca8 aca10* and *aca4 aca11* had significantly more *PR1* expression compared with the wild type under NH_4_NO_3_ supplement (Fig. 7A).

We further examined the effect of the anion supplement on disease resistance in the *aca8 aca10* mutant. Plants were grown in hydroponic solution with or without the NH_4_NO_3_ supplement for 4 weeks and dipping-inoculated with *Pst* DC3000. After 3 d of growth, pathogen growth was quantified in the wild type and the mutant. The *aca8 aca10* mutant was slightly more resistant than the wild type to *Pst* DC3000 when grown in standard solution (Fig. 7B). With the anion supplement, resistance was greatly reduced in both the wild type and the *aca8 aca10* mutant, and the *aca8 aca10* mutant was more resistant than the wild type even under anion-supplemented condition (Fig. 7B).

## Discussion

In this study, we analysed the contribution of four PM-localized Ca^2+^ ATPases, ACA8, ACA10, ACA12 and ACA13, to plant growth and immunity. Mutant combinations of their genes revealed overlapping functions between multiple *ACA* pairs, including *ACA8–ACA10*, *ACA12–ACA13*, *ACA8–ACA12*, *ACA8–ACA13*, *ACA10–ACA12*, and *ACA10–ACA13* in plant growth, stomatal response, and resistance to bacterial pathogens (see Supplementary Fig. S2). They indicate not only the importance of each ACA in development and environmental responses but also a differential contribution of these ACAs in rosette growth, inflorescence stem elongation, seed setting, stomatal closure response, and disease resistance. We demonstrate for the first time that *ACA12* and *ACA13* play broader roles in development and environmental responses than previously thought. We found that *ACA10* plays a major role in plant immunity, and *ACA8*, *ACA12*, and *ACA13* also play roles in immunity in the absence of *ACA10* (Supplementary Fig. S2E). In addition, *ACA10* takes the largest role in vegetative growth, followed by *ACA8* and then *ACA12* and *ACA13* (Supplementary Fig. S2A, B).

The developmental function of *ACAs*, especially *ACA9* and *ACA7*, was demonstrated in pollen germination (Schiøtt *et al.*, 2004; Lucca and León, 2012), and the stigmatic function of *ACA13* was also demonstrated (Iwano *et al.*, 2014). Here we find that the *aca10 aca13* double mutant, but not any of the other double mutants, led to no seed production (Fig. 3D). This could be due to a failure in gamete formation, pollination, fertilization, and/or embryogenesis. Detailed anatomical and physiological analysis will reveal which processes and which cells these ACAs could play critical roles in. In any case, these data indicate an overlapping role of *ACAs* in one or several processes and an essential function of calcium signaling in reproductive growth.

*ACA10* and *ACA8* have, in general, a larger role in leaf growth and immunity than *ACA12* and *ACA13*. This appears to largely result from differential expression pattern of these four genes. *ACA8* and *ACA10* expression is much higher than *ACA12* and *ACA13* in the vegetative rosette, which likely accounts for the severe growth defect of the *aca8 aca10* double mutant compared with the *aca12 aca13* double mutant (see Supplementary Table S2A). It is also interesting that *ACA13* expression was up-regulated in the rosette when *ACA10* and *ACA8* are knocked out (Fig. 1B), suggesting that there could be compensation among the members. Indeed, the function of *ACA12* and *ACA13* became apparent when the *ACA10* and *ACA8* functions were knocked out. In pollen, *ACA13* has a much higher expression than in leaves and roots, and the level is comparable to that of *ACA10* (Supplementary Table S2A). This is correlated with the major role it plays among the four *ACA*s in seed production. In addition, expression of *ACA12* has a higher induction in response to pathogens than the other three *ACA* genes, while *ACA10* has a much higher expression overall than the others (Supplementary Table S2B), suggesting a possible fine tuning of biotic responses through regulation of *ACA* gene expression. Interestingly, none of the four genes has a drastic change in the expression in response to abiotic stress. The roles of these genes in abiotic stress responses are largely uncharacterized. When they are characterized, the dynamics of calcium signaling might be found to be different in biotic and abiotic responses.

The differential role of these ACAs might also result from their different biochemical properties (Bonza and De Michelis, 2011). Relative expression levels are not always correlated with the relative roles of these ACAs. *ACA12* and *ACA13* have much higher expression in guard cells than other tissues and their expression levels are comparable to or higher than *ACA8* and *ACA10* (Supplementary Table 1A), but *ACA8* and *ACA10* play a more critical role in guard cell response to calcium and ABA signals (Fig. 5). The regulation of ACA10 and ACA8 proteins is thought to be different from that of ACA12 and ACA13 proteins. The activities of ACA10 and ACA8 are calcium regulated through the auto-inhibitory domain that overlaps with the calmodulin binding motif (Tidow *et al.*, 2012). These features confer regulation on ACA10 and ACA8 activities by calcium, CaM and the interacting protein BON1 (Yang *et al.*, 2017). In contrast, the activity of ACA12 is deregulated, that is, it does not contain the auto-inhibitory domain (Limonta *et al.*, 2014). Sequence alignment suggests that ACA13 is also likely to be deregulated (Limonta *et al.*, 2014). The lower expression in the vegetative tissues of *ACA12* and *ACA13* might provide a low constitutive calcium pump activity irrespective of calcium status in the cell.

The *aca10 aca13 aca8/+* triple mutants exhibited lethality at bolting (Fig. 2A). Two scenarios, alone or in combination, could contribute to the lethality. One is that ACAs have essential functions in cell physiology such as calcium homeostasis, and shoot apical meristem cells cannot transit from vegetative growth to reproductive growth (Bonza *et al.*, 2016; Zhu, 2016). The other scenario is that the shoot apical meristem cells have heightened defense responses leading to cell death. This would be similar to the mutant of BON1 which interacts with and potentially activates ACA8 and ACA10 (Yang *et al.*, 2017). The progressive loss of the *BON1* family members results in progressively increased disease resistance and ultimately lethality that can be suppressed by inhibition of defense response up-regulation (Yang *et al.*, 2006; Li *et al.*, 2009).

Calcium signaling is critical for the closing and opening of stomata (Kudla *et al.*, 2010; Zou *et al.*, 2010). *ACA8* and *ACA10* have been shown to modulate stomatal closure in response to calcium, ABA, and pathogens (Yang *et al.*, 2017). Here we revealed a role of both *ACA12* and *ACA13* in stomatal closure when the *ACA8* function is abolished. Because ACA8 and ACA10 are important for calcium signature generation and calcium homeostasis (Yang *et al.*, 2017), it is likely ACA12 and ACA13 also participate in generating the calcium signature and impact the steady level of calcium (Iwano *et al.*, 2014; Limonta *et al.*, 2014). Intriguingly, stomata of *aca8 aca10*, *aca8 aca10 aca12*, and *aca8 aca10 aca13/+* mutants had a smaller aperture after treatment with opening buffer (Fig. 5), suggesting that they are defective in stomatal opening as well. These data suggest that the ACAs are important for both opening and closure responses in guard cells.

The growth defects observed in the *ACA* mutant combinations are likely partially due to up-regulation of defense responses. The reduced rosette size and inflorescence stem observed in multiple *aca* double mutants at 22°C are greatly reduced at 28°C (Figs 2, 3). Elevated temperature could inhibit disease resistance gene-mediated defense responses and the associated growth inhibition (Hua, 2013). It is possible that up-regulation of defense responses in the *aca* mutant combinations contributed greatly to the growth defects. This was demonstrated for the *aca10* and *aca8 aca10* mutants where inhibition of defense responses rescued their growth defect (Yang *et al.*, 2017). The lesion phenotype of the *aca4 aca11* double mutant could be suppressed by high concentrations of various anions, such as NO_3_^−^ (Boursiac *et al.*, 2010). Here we found that the narrow leaf phenotype of *aca8 aca10* could also be largely suppressed by anion supplements (Fig. 6). Anion supplements reduced the accumulation of *PR1* in all plants including Col-0, *aca8 aca10*, and *aca4 aca11* (Fig. 7A). Despite the greatly reduced *PR1* expression in general, the *aca8 aca10* mutant still exhibited a higher resistance to *Pst* DC3000 compared with the wild type under standard hydroponic conditions or anion supplemented conditions (Fig. 7B). This suggests that anion suppression works downstream of the early signaling pathway and upstream of the transcriptional response of the *PR1* gene. Therefore, perturbation of ACA8/10 function, similarly to ACA4/11 function, induces heightened disease resistance as well as up-regulation of *PR1*, which can be differentially influenced by the anion supplement.

Interestingly, we found that both the wild type and the mutants had higher *PR1* expression under standard solution than solution supplemented with NH_4_NO_3_ (Fig. 7A). The NH_4_NO_3_ supplement also alleviated the growth defects but not the enhanced resistance in the *aca* mutants. Previous study showed that the anion supplements did not suppress the accumulation of reactive oxygen species and salicylic acid but prevented the accumulation of *PR1* in the *aca4 aca11* mutant (Boursiac *et al.*, 2010). Therefore, anion balance likely has a large impact on the expression of *PR1* even in the absence of pathogen invasion. It may also affect expression of other genes directly related to plant growth independent of disease resistance.

While we have evaluated the roles of four PM-localized ACA genes, *ACA8*, *ACA10*, *ACA12*, and *ACA13*, in a number of processes through genetic characterizations, how they exert their regulation on these processes is not yet determined. Future studies should reveal how they individually and combinatorically affect the basal levels of calcium in specific cell types and tissues as well as the kinetics of calcium transients in response to environmental changes. That knowledge will contribute to the ultimate understanding of the regulation of calcium signals to achieve a fine-tuned and highly adaptive calcium signaling system in plant growth and environmental responses.

## Supplementary data

Supplementary data are available at *JXB* online.

Fig. S1. Growth phenotypes of the full set of *aca* mutants in the seedling stage.

Fig. S2. Diagram of genetic interaction of the four *ACA* genes.

Table S1. List of all oligonucleotides used in this study.

Table S2. Expression level of four PM *ACA* genes.

Supplementary Figures and Tables
