# Supplementary material for: Overlapping and differential roles of plasma membrane calcium ATPases in Arabidopsis growth and environmental responses
Source: J Exp Bot. 2018 Mar 1;69(10):2693–703. doi: 10.1093/jxb/ery073 (PMC5920303; doi:10.1093/jxb/ery073)
Supplement: Supplementary Figures and Tables [file ery073_suppl_supplementary_figures_and_tables.pdf]

Supplemental Table 1. Expression levels of four PM ACA genes.

A, Expression in different tissues. B-C, Expression under biotic stresses (B) and abiotic stresses (C).
Data was acquired from the RNA-seq and microarray data (Winter et al., 2007) from eFP Browser at TAIR.

A

Tissue \ Gene	ACA8	ACA10	ACA12	ACA13
Dry seed	566	386	23	26
Imbibed seed, 24 h	290	201	3	6
1st Node	263	307	7	10
Cauline Leaf	102	668*	54	10
Cotyledon	93	375	26	24
Root	355	162	20	16
Entire Rosette After Transition to Flowering	171	259	23	15
Flower Stage 9	300	157	5	7
Flower Stage 10/11	310	187	7	10
Flower Stage 12	223	187	17	148
Flower Stage 15	176	300	57	150
Flower Stage 12, Carpels	375	123	10	9
Flower Stage 12, Petals	182	158	10	38
Flower Stage 12, Sepals	112	518	36	91
Flower Stage 12, Stamens	182	183	10	1333
Flower Stage 15, Carpels	352	211	7	28
Flower Stage 15, Petals	97	310	53	23
Flower Stage 15, Sepals	56	575	207	282
Flower Stage 15, Stamen	111	278	27	1484
Flowers Stage 15, Pedicels	167	311	12	25
Leaf 1 + 2	105	159	8	14
Leaf 7, Petiole	209	251	17	9
Leaf 7, Distal Half	179	382	22	20
Leaf 7, Proximal Half	193	339	21	11
Hypocotyl	364	222	18	10
Root	477	155	19	18
Rosette Leaf 2	90	601	44	24
Rosette Leaf 4	152	491	26	23
Rosette Leaf 6	162	351	20	23
Rosette Leaf 8	189	336	11	13
Rosette Leaf 10	193	265	20	10
Rosette Leaf 12	221	214	17	11
Senescing Leaf	126	631	356	17
Shoot Apex, Inflorescence	348	141	6	2
Shoot Apex, Transition	263	118	8	3
Shoot Apex, Vegetative	185	124	9	10
Stem, 2nd Internode	252	509	19	8
Mature Pollen	110	31	13	27
Seeds Stage 3 w/ Siliques	126	133	13	31
Seeds Stage 4 w/ Siliques	221	104	9	27
Seeds Stage 5 w/ Siliques	234	114	12	46
Seeds Stage 6 w/o Siliques	303	90	7	65
Seeds Stage 7 w/o Siliques	438	92	11	78
Seeds Stage 8 w/o Siliques	509	72	14	36
Seeds Stage 9 w/o Siliques	535	87	21	56
Seeds Stage 10 w/o Siliques	434	79	13	44
Vegetative Rosette	152	124	9	7
Guard cells	137	400	1868	387

*Shade denotes the highest expression that is more than two fold of the second highest among the four genes or the expression of *ACA12* or *ACA13* is comparable to that of ACA10 or ACA8.

B

Biotic Stress / Gene	*Botrytis cinerea*		*Pseudomonas syringae*		flg22		*Phytophthora infestans*		*Erysiphe orontii*	
	Control (C)	Treatment (T)	C	T	C	T	C	T	C	T
ACA8	159	173	192	69**	296	176	114	78	66	153*
ACA10	316	508	263	551	157	326	205	289	158	190
ACA12	66	751	35	272	25	171	23	85	25	23
ACA13	10	16	14	67	25	22	14	7	5	4

*Gray shade denotes treatment expressions that is more than two fold than the control expression.
**Underline denotes treatment expression that is much reduced compared to the control expression.

C

Abiotic Stress / Gene	Cold	Osmotic	Salt	Drought	Genotoxic	Oxidative	UV-B	Wounding	Heat
ACA8	+	-	-	-	-	-	-	-	-
ACA10	-	-	-	-	-	-	+	-	-
ACA12	-	-	-	-	-	-	+	-	-
ACA13	-	+	-	-	-	-	-	-	-

'+' : upregulate; '-' : no response.

Supplemental Table 2 List of all oligonucleotides used in this study.

T-DNA insertion alleles of *ACA8*, *ACA10*, *ACA12*, and *ACA13* were PCR-amplified using the T-DNA specific primers GABI-LB (*aca8*, *aca10*), SALK-LBb1.3 (*aca12*), or SAIL-LB1 (*aca13*), in combination with a gene specific primer aca8-RP (*aca8)*, aca10-RP (*aca10)*, aca12RP *(aca12),* or aca13RP (*aca13*). The wild type alleles of these genes were amplified with the above gene-specific primer with another gene specific primer: aca8-LP (*ACA8*), aca10-LP (*ACA10*), aca12LP (*ACA12*), or aca13LP (*ACA13*).

Primer Name	Sequence (5'-3')
aca8-LP	GAGTTTCTTCACCATTGTCT
aca8-RP	GACATAGTGGTGGGTGATGT
aca10-LP	GAACCTGACGCCAATAGTG
aca10-RP	CAAGACCATGTCATACTGC
aca12LP	CTTCATGGATTGACTCTTGGC
aca12RP	TTCCTGCCATTGATCAAGAAC
aca13LP	GACACTAACGAGCAAACTCCG
aca13RP	TCTCGCCGTGAAAATGTTATC
GABI-LB	TTGGACGTGAATGTAGACAC
SALK-LBb1.3	ATTTTGCCGATTTCGGAAC
SAIL-LB1	GCCTTTTCAGAAATGGATAAATAGCCTTGCTTCC
PR1-QRT-F	CGAGAAGGCTAACTACAACTACG
PR1-QRT-R	ACACCTCACTTTGGCACATC
ACT2 F	CACCACCTGAAAGGAAGTACAG
ACT2 R	TGGACCTGCCTCATCATACT

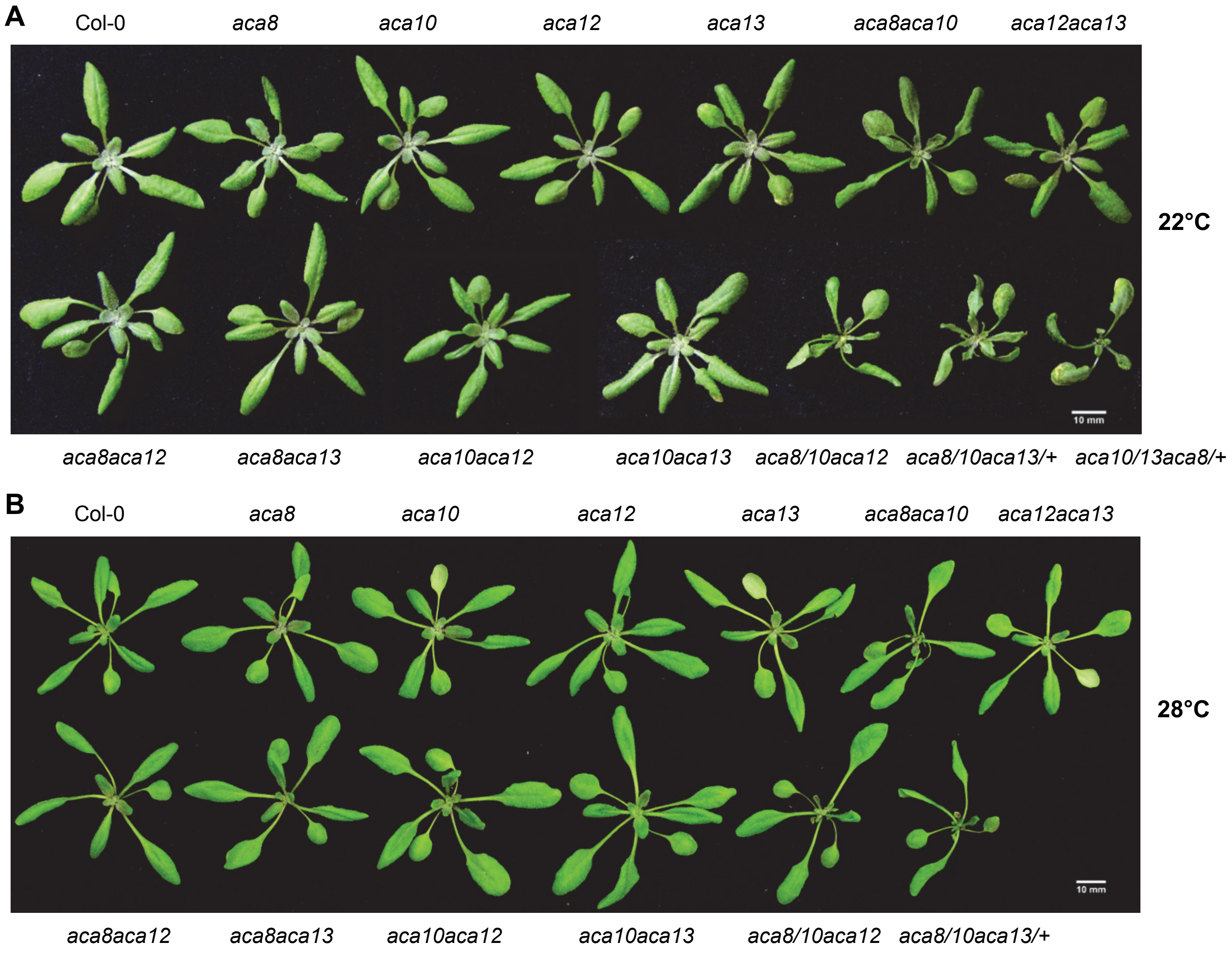

Supplemental Figure S1 Growth phenotypes of the full set of *aca* mutants in the seedling stage. Shown are growth phenotypes under constant light for 18 days at 22°C (A) or for 15 days at 28°C (B). Figure 2 shows a selected set of these plants.

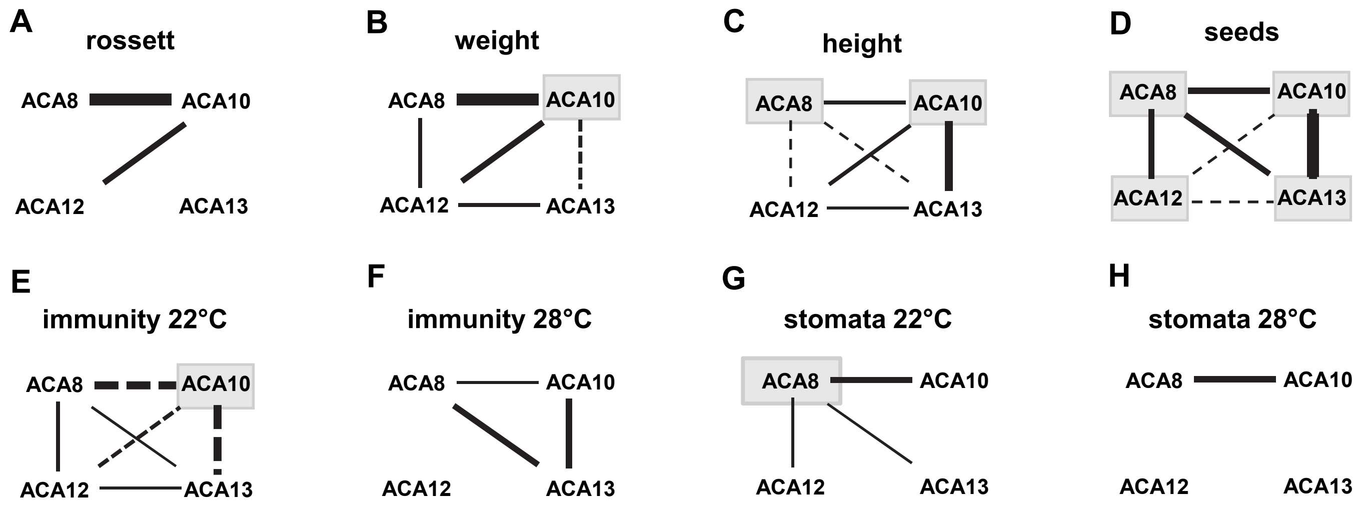

Supplemental Figure S2 Diagram of interaction of the four ACA genes.

Diagram illustrating the genetic interaction of the four ACA genes in regulating rosette size (A), weight (B), inflorescent height (C), seeds setting (D), disease resistance at 22°C (E), disease resistance at 28°C (F), stomata closure response at 22°C (G) and stomata closure response at 28°C (H). Solid lines represent the existence of an enhanced mutant phenotype in the double mutants compared to the single mutants. Dotted lines indicate the double mutant has the same phenotype as one of the single mutant. The width of the line represents the relative severity of the double mutant phenotype, with wider line indicating more severe phenotype. Gray box indicates that the single mutant of the gene shows a defect.
